# 晚期非小细胞肺癌患者应用免疫治疗后出现甲状腺功能异常与疗效的相关性分析

**DOI:** 10.3779/j.issn.1009-3419.2023.106.09

**Published:** 2023-05-20

**Authors:** WANG Yibo, WANG Xinjuan, CHENG Lin, ZHANG Guojun

**Affiliations:** 450000 郑州，郑州大学第一附属医院呼吸内科; Department of Pulmonary Medicine, The First Affiliated Hospital of Zhengzhou University, Zhengzhou 450000, China

**Keywords:** 肺肿瘤, 免疫检查点抑制剂, 免疫相关不良事件, 甲状腺功能, 疗效, Lung neoplasms, Immune checkpoint inhibitors, Immune-related adverse events, Thyroid function, Efficacy

## Abstract

**背景与目的** 甲状腺功能异常（thyroid function abnormality, TFA）是晚期非小细胞肺癌（non-small cell lung cancer, NSCLC）患者常见的免疫治疗相关不良内分泌事件，但TFA发生的危险因素及其与患者疗效的关系尚不完全清楚。本研究旨在探讨晚期NSCLC患者应用免疫治疗后出现TFA的危险因素及其与疗效的关系。 **方法** 回顾性收集2019年7月1日-2021年6月31日就诊于郑州大学第一附属医院的晚期NSCLC患者的临床资料，应用χ²检验及多因素Logistic回归探究TFA发生的影响因素。绘制Kaplan-Meier曲线，采用Log-rank检验进行组间比较，应用单因素及多因素Cox分析探究疗效的影响因素。**结果** 共有86例（43.10%）患者发生TFA，Logistic回归分析发现美国东部肿瘤协作组体能状态（Eastern Cooperative Oncology Group performance status, ECOG PS）、胸腔积液、乳酸脱氢酶（lactic dehydrogenase, LDH）是TFA发生的影响因素（P<0.05）；TFA组患者相比于甲状腺功能正常组患者的中位无进展生存期（progression-free survival, PFS）显著延长（19.0 个月 vs 6.3 个月，P<0.001），且TFA组的客观缓解率（objective response rate, ORR）（65.1% vs 28.9%, P<0.001）和疾病控制率（disease control rate, DCR）（100.0% vs 92.1%, P=0.020）均优于甲状腺功能正常组。Cox回归分析示ECOG PS、LDH、细胞角蛋白19片段（cytokeratin 19 fragment, CYFRA21-1）、TFA是疗效的影响因素（P<0.05）。**结论** ECOG PS、胸腔积液、LDH可能是TFA发生的影响因素，TFA可能是免疫治疗疗效的影响因素，接受免疫治疗后发生TFA的晚期NSCLC患者可能有更好的疗效。

## Abstract (en)

**Background and objective** Thyroid function abnormality (TFA) is one of the common adverse reactions in patients with advanced non-small cell lung cancer (NSCLC) treated with immunotherapy, but the risk factors of TFA and its relationship with efficacy are not completely clear. The purpose of this study was to explore the risk factors of TFA and its relationship with efficacy in patients with advanced NSCLC after immunotherapy. **Methods** The general clinical data of 200 patients with advanced NSCLC in The First Affiliated Hospital of Zhengzhou University from July 1, 2019 to June 31, 2021 were collected and analyzed retrospectively. χ² test and multivariate Logistic regression were used to explore the risk factors of TFA. Kaplan-Meier curve was drawn and Log-rank test was used for comparison between groups. Univariate and multivariate Cox analysis was used to explore the efficacy factors. **Results** A total of 86 (43.0%) patients developed TFA. Logistic regression analysis showed that Eastern Cooperative Oncology Group Performance Status (ECOG PS), pleural effusion and lactic dehydrogenase (LDH) were factors influencing TFA (P<0.05). Compared with normal thyroid function group, the median progression-free survival (PFS) of patients in the TFA group was significantly longer (19.0 months vs 6.3 months, P<0.001), and the objective response rate (ORR) (65.1% vs 28.9%, P=0.020) and disease control rate (DCR) (100.0% vs 92.1%, P=0.020) of the TFA group were better than those of the normal thyroid function group. Cox regression analysis showed that ECOG PS, LDH, cytokeratin 19 fragment (CYFRA21-1) and TFA were factors influencing prognosis (P<0.05). **Conclusion** ECOG PS, pleural effusion and LDH may be risk factors affecting the occurrence of TFA and TFA may be a predictor of the efficacy of immunotherapy. Patients with advanced NSCLC who have TFA after immunotherapy may obtain better efficacy.

国家癌症中心统计^[1]^显示2016年我国新增癌症病例与新增癌症死亡患者共647.7万例，无论发病人数还是死亡人数，肺癌均占据第一。肺癌依照病理类型，可以分为非小细胞肺癌（non-small cell lung cancer, NSCLC）和小细胞肺癌（small cell lung cancer, SCLC），其中NSCLC占80%-85%^[2]^。对驱动基因阳性的晚期NSCLC患者，目前以靶向治疗为主，但对驱动基因阴性的患者，传统治疗以化疗为主，近年来以细胞程序性死亡受体1（programmed cell death 1, PD-1）/PD-1配体（programmed cell death ligand 1, PD-L1）为代表的免疫检查点抑制剂（immune checkpoint inhibitors, ICIs）的兴起为驱动基因阴性的晚期NSCLC的治疗带来了希望。但免疫治疗在为患者带来显著疗效的同时也会引起一系列免疫相关不良事件（immune-related adverse events, irAEs），可累及皮肤、呼吸道、消化道及内分泌等多个系统^[3,4]^，所有级别irAEs的发生率为65%-76%^[5]^。甲状腺功能异常（thyroid function abnormality, TFA）是常见的免疫相关不良反应之一，发生率约为25%^[6]^。Wu等^[7]^发现甲状腺抗体及促甲状腺激素（thyroid stimulating hormone, TSH）水平与TFA的发生有关，但TFA与一般临床资料的关系尚不清楚。既往研究^[8,9]^发现TFA与肾癌、黑色素瘤等患者获得更好的预后相关，但纳入研究的癌症类型及治疗线数不一。本研究旨在探讨晚期NSCLC患者一线接受免疫治疗后发生TFA的危险因素及其与疗效的关系。

## 1 资料与方法

### 1.1 研究对象

回顾性收集2019年7月1日-2021年6月31日就诊于郑州大学第一附属医院的晚期NSCLC患者的临床资料，其治疗方案为一线应用免疫治疗。本研究的纳入标准为：（1）年龄为18岁以上的成人患者；（2）组织病理确诊为NSCLC，肿瘤原发灶-淋巴结-转移（tumor-node-metastasis, TNM）分期为IIIB期、IIIC期或IV期的患者；（3）至少接受过2个周期免疫治疗；（4）无严重的心脏、脑和肾脏等重要脏器合并症。排除标准：（1）合并其他部位的原发肿瘤；（2）全身病灶均为不可测量病灶；（3）既往合并甲状腺功能亢进、甲状腺功能减退等甲状腺相关疾病；（4）临床资料、实验室检查、影像学等病历资料不完整。本研究得到郑州大学第一附属医院伦理委员会批准（No.2023-KY-0390-002）。

### 1.2 一般资料

基于郑州大学第一附属医院电子病历系统进行检索，收集患者的临床资料，如年龄、性别、吸烟史、免疫治疗药物、治疗方案、美国东部肿瘤协作组体能状态（Eastern Cooperative Oncology Group Performance Status, ECOG PS）等。检查检验信息：常规抽血指标（血常规、生化、肿瘤标志物等）、病理类型、肿瘤分化程度、肿瘤部位、肿瘤最大直径、是否合并远处转移、TNM分期、PD-L1表达情况等。

### 1.3 甲状腺功能检测

甲状腺功能由我院核医学科采用化学发光免疫分析（chemiluminescence analysis, CLIA）进行检测，检测设备及厂家为：贝克曼库尔特Unicel DxI 800免疫分析系统。甲状腺功能参考值：游离三碘甲状腺原氨酸（free triiodothyronine, FT3）：3.28 pmol/L-6.47 pmol/L；游离甲状腺激素（free thyroxine, FT4）：7.9 pmol/L-18.4 pmol/L；TSH：0.34 μIU/mL-5.6 μIU/mL，记录第一次接受免疫治疗前的基线FT4、FT3、TSH水平，是否发生TFA、TFA的类型以及发生时间。TFA类型划分标准：甲状腺功能减退：TSH增高，FT4、FT3降低；亚临床甲状腺功能减退：仅有TSH增高，FT4、FT3正常；TSH降低，FT3、FT4降低定义为中枢性甲状腺功能减退。甲状腺功能亢进：TSH降低，FT4、FT3增高；亚临床甲状腺功能亢进：仅有TSH降低，FT4、FT3正常。一过性甲状腺功能异常定义为：FT3、FT4异常，TSH正常。TFA发生时间定义为从开始接受免疫治疗到首次发现TFA的时间。

### 1.4 疗效评估及随访

通过电子病历系统及电话进行随访。通过胸部计算机断层扫描（computed tomography, CT）、全身骨显像以及核磁共振成像（magnetic resonance imaging, MRI）等影像学检查进行疗效评估。根据实体肿瘤疗效评估标准1.1版（Response Evaluation Criteria in Solid Tumors Version 1.1, RECIST v1.1）^[10]^每6周-8周进行疗效评估，分为完全缓解（complete response, CR）、部分缓解（partial response, PR）、疾病稳定（stable disease, SD）和疾病进展（progressive disease, PD）。将第一次接受免疫治疗开始一直到PD或因任何原因死亡的时间定义为无进展生存期（progression-free survival, PFS）。客观缓解率（objective response rate, ORR）=（CR+PR）例数/总例数×100%。疾病控制率（disease control rate, DCR）=（CR+PR+SD）例数/总例数×100%。本研究主要观察终点为根据RECIST v1.1评估的PFS，次要观察终点为ORR、DCR。

### 1.5 统计学分析

应用SPSS 25.0软件和X-Tile软件3.6.1版进行数据分析与绘图。采用χ²检验及多因素二元Logistic回归模型分析患者一般临床特征与TFA的关系。选择Kaplan-Meier法进行生存分析，采用Log-rank检验进行组间比较。应用X-Tile识别PFS二分类变量的最佳分界值。应用单因素及多因素Cox比例风险模型分析疗效的影响因素。P<0.05为差异有统计学意义，P值为双侧检验。

## 2 结果

### 2.1 患者临床特征

共纳入200例晚期NSCLC患者（表1）。男性患者159例（79.5%）；年龄在65岁以下122例（61.0%）；133例（66.5%）患者有吸烟史；161例（80.5%）患者ECOG PS评分为0分-1分，39例（19.5%）为2分；鳞癌患者113例（56.5%），87例（43.5%）为非鳞NSCLC；140例（70.0%）患者为IV期；发生TFA的患者有86例（43.0%），114例（57.0%）没有发生TFA。治疗方案：本研究中200例患者均为一线即应用免疫治疗，159例（79.5%）患者为化疗加免疫治疗，18例（9.0%）患者为免疫治疗加抗血管药物治疗，15例（7.5%）患者为化疗加免疫治疗加抗血管药物治疗，8例（4.0%）为免疫单药治疗。应用的免疫治疗药物中，186例（93.0%）应用PD-1单抗，14例（7.0%）应用PD-L1单抗。疗效：200例患者中，均未达到CR，达到PR患者有89例（44.5%），达到SD患者有102例（51.0%），达到PD患者有9例（4.5%）。

**表1:** 200例晚期NSCLC患者的临床资料

Characteristic		n	Percentage (%)
Age (yr)	<65	122	61.0
	≥65	78	39.0
Gender	Female	41	20.5
	Male	159	79.5
ECOG PS	0-1	161	80.5
	2	39	19.5
Smoking history	Yes	133	66.5
	No	67	33.5
Histology	Squamous carcinoma	113	56.5
	Non-squamous carcinoma	87	43.5
TNM stage	III	60	30.0
	IV	140	70.0
Treatment plan	Immunotherapy plus chemotherapy	159	79.5
	Immunotherapy plus anti-vascular therapy	18	9.0
	Immunotherapy plus chemotherapy plus anti-vascular therapy	15	7.5
	Immunotherapy	8	4.0
PD-L1 expression	Positive	81	40.5
	Negative	35	17.5
	Unknown	84	42.0
Curative effect	PR	89	44.5
	SD	102	51.0
	PD	9	4.5
Uric acid (μmol/L)	≤213	46	23.0
	>213	154	77.0
CYFRA21-1 (ng/mL)	≤2.35	38	19.0
	>2.35	162	81.0
NLR	≤4.86	160	80.0
	>4.86	40	20.0
LDH (U/L)	≤245	138	69.0
	>245	62	31.0
TFA	Yes	86	43.0
	No	114	57.0
PD-1 inhibitors	Camrelizumab	118	59.0
	Tislelizumab	22	11.0
	Sintilimab	15	7.5
	Toripalimab	13	6.5
	Penpulimab	10	5.0
	Pembrolizumab	8	4.0
PD-L1 inhibitors	Atezolizumab	6	3.0
	Durvalumab	3	1.5
	Sugemalimab	3	1.5
	Adebrelimab	2	1.0

NSCLC: non-small cell lung cancer; ECOG PS: Eastern Cooperative Oncology Group performance status; TNM: tumor-node-metastasis; PR: partial response; SD: stable disease; PD: progressive disease; CYFRA21-1: cytokeratin 19 fragment; NLR: neutrophil-to-lymphocyte ratio; LDH: lactic dehydrogenase; TFA: thyroid function abnormality; PD-1: programmed cell death 1; PD-L1: programmed cell death ligand 1.

### 2.2 TFA及危险因素分析

共有86例（43.0%）患者发生TFA，TFA发生的中位时间是6.4个月，其中亚临床甲状腺功能减退有27例（31.4%），亚临床甲状腺功能亢进有27例（31.4%），甲状腺功能减退有11例（12.8%），甲状腺功能亢进有7例（8.1%），一过性甲状腺功能异常有12例（14.0%），中枢性甲状腺功能减退有2例（2.3%）。TFA的严重程度参考美国癌症中心常见毒副反应标准，86例TFA患者中，严重程度为1级的有66例（76.7%），2级有20例（23.3%），未观察到2级以上的TFA发生。将单因素分析（表2）中有意义的ECOG PS（P<0.001）、胸腔积液（P=0.006）、TNM（P=0.025）、尿酸（P=0.050）、乳酸脱氢酶（lactic dehydrogenase, LDH）（P=0.040）纳入多因素二元Logistic回归模型，结果（表3）显示影响TFA发生的独立预测因子为ECOG PS[比值比（odds ratio, OR）=11.930，95%置信区间（confidence interval, CI）：3.438-41.401，P<0.001]、胸腔积液（OR=3.209, 95%CI: 1.202-8.564, P=0.02）和LDH（OR=2.007, 95%CI: 1.008-3.995, P=0.047）。

**表2:** 200例晚期NSCLC患者TFA单因素分析

Characteristic	n	TFA group	Euthyroidism group	χ²	P
Age (yr)				2.632	0.105
≥65	78	28 (35.9%)	50 (64.1%)		
<65	122	58 (47.5%)	64 (52.5%)		
Gender				1.649	0.199
Male	159	72 (45.3%)	87 (54.7%)		
Female	41	14 (34.1%)	27 (65.9%)		
Smoking history				0.723	0.395
Yes	133	60 (45.1%)	73 (54.9%)		
No	67	26 (38.8%)	41 (61.2%)		
Histology				0.965	0.326
Squamous carcinoma	113	52 (46.0%)	61 (54.0%)		
Non-squamous carcinoma	87	34 (39.1%)	53 (60.9%)		
ECOG PS				24.641	<0.001
0-1	161	83 (51.6%)	78 (48.4%)		
2	39	3 (7.7%)	36 (92.3%)		
Pleural effusion				7.654	0.006
Yes	33	7 (21.2%)	26 (78.8%)		
No	167	79 (47.3%)	88 (52.7%)		
TNM stage				5.036	0.025
III	60	33 (55.0%)	27 (45.0%)		
IV	140	53 (37.9%)	87 (62.1%)		
Uric acid (μmol/L)				3.848	0.050
≤213	46	14 (30.4%)	32 (69.6%)		
>213	154	72 (46.8%)	82 (53.2%)		
LDH (U/L)				4.230	0.040
≤245	138	66 (47.8%)	72 (52.2%)		
>245	62	20 (32.3%)	42 (67.7%)		
Treatment plan				1.851	0.604
Immunotherapy plus chemotherapy	159	71 (44.7%)	88 (55.3%)		
Immunotherapy plus anti-vascular therapy	18	8 (44.4%)	10 (55.6%)		
Immunotherapy plus chemotherapy plus anti-vascular therapy	15	4 (26.7%)	11 (73.3%)		
Immunotherapy	8	3 (37.5%)	5 (62.5%)		

**表3:** 多因素Logistic分析TFA发生的相关影响因素

Variable	β	SE	Wald	OR	95%CI	P
ECOG PS	2.479	0.635	15.249	11.930	3.438-41.401	<0.001
Pleural effusion	1.166	0.501	5.419	3.209	1.202-8.564	0.020
TNM stage	0.161	0.349	0.213	1.175	0.593-2.330	0.644
Uric acid	-0.402	0.402	1.000	0.669	0.304-1.471	0.317
LDH	0.697	0.351	3.933	2.007	1.008-3.995	0.047
Constant	-0.219	0.442	0.245	0.804	-	0.621

SE: standard error; OR: odds ratio; CI: confidence interval.

### 2.3 生存分析

200例晚期NSCLC患者的中位随访时间为21.9个月，中位PFS为11.2个月。发生TFA的患者PFS显著延长，中位PFS为19.0个月，甲状腺功能正常组中位PFS为6.3个月，差异具有统计学意义（P<0.001，图1A）。200例患者总体ORR为44.5%，DCR为95.5%；TFA组的ORR（65.1% vs 28.9%, P<0.001）和DCR（100.0% vs 92.1%, P=0.02）均优于甲状腺功能正常组。

**图1:**
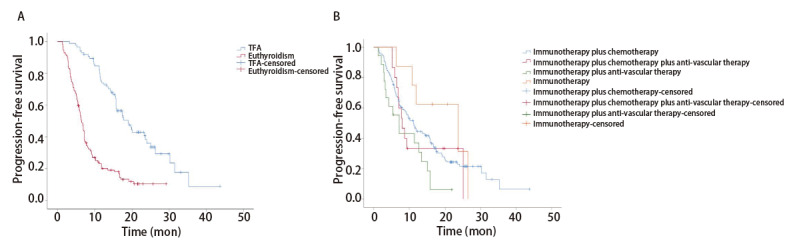
无进展生存期生存曲线。A：甲状腺功能正常组和TFA组患者；B：不同治疗组。

对不同治疗组PFS进行分析，单药免疫组中位PFS为23.6个月，化疗+免疫组中位PFS为11.2个月，化疗+免疫+抗血管组中位PFS为8.0个月，免疫+抗血管组中位PFS为7.2个月；4个治疗组总体分析发现差异无统计学意义（P=0.064），两两比较发现，化疗+免疫组和免疫+抗血管组（P=0.021）、免疫+抗血管组和单药免疫组（P=0.010）的差异具有统计学意义（表4，图1B）。

**表4:** 不同治疗模式患者的PFS的比较

Treatment model					mPFS (mon)	P
Immunotherapy plus chemotherapy vs Immunotherapy plus anti-vascular therapy					11.2 vs 7.2	0.021
Immunotherapy plus chemotherapy vs Immunotherapy plus chemotherapy plus anti-vascular therapy					11.2 vs 8.0	0.841
Immunotherapy plus chemotherapy vs Immunotherapy					11.2 vs 23.6	0.260
Immunotherapy plus anti-vascular therapy vs Immunotherapy					7.2 vs 23.6	0.010
Immunotherapy plus anti-vascular therapy vs Immunotherapy plus chemotherapy plus anti-vascular therapy					7.2 vs 8.0	0.183
Immunotherapy plus chemotherapy plus anti-vascular therapy vs Immunotherapy					8.0 vs 23.6	0.112
mPFS: median progression-free survival.						

对200例患者的PFS进行单因素和多因素分析Cox回归分析（表5），单因素分析结果显示，ECOG PS评分0分-1分、没有骨转移、没有其他远处转移、TNM分期为III期、尿酸>213 μmol/L、LDH≤245 U/L、细胞角蛋白19片段（cytokeratin 19 fragment, CYFRA21-1）>2.35 ng/mL、中性粒细胞与淋巴细胞比值（neutrophil-to-lymphocyte ratio, NLR）≤4.86、发生TFA均与较长的PFS有关（P<0.05）；将这些因素纳入多因素分析，结果显示ECOG PS评分0分-1分、LDH≤245 U/L、CYFRA21-1>2.35 ng/mL、发生TFA与较长的PFS有关。

**表5:** 200例晚期NSCLC患者PFS单因素与多因素Cox回归分析

Characteristic	Univariate analysis			Multivariate analysis	
	HR (95%CI)	P		HR (95%CI)	P
Age (<65 yr vs ≥65 yr)	0.874 (0.624-1.224)	0.433		-	-
Gender (Female vs Male)	1.389 (0.951-2.030)	0.089		-	-
ECOG PS (0-1 vs 2)	3.498 (2.343-5.221)	<0.001		1.953 (1.259-3.031)	0.003
Smoke (Yes vs No)	1.139 (0.812-1.598)	0.451		-	-
Histology (Squamous vs Non-squamous)	1.025 (0.791-1.328)	0.851		-	-
Bone metastases (Yes vs No)	0.570 (0.397-0.819)	0.002		0.744 (0.498-1.111)	0.148
Other metastases (Yes vs No)	0.681 (0.463-1.001)	0.050		0.928 (0.612-1.408)	0.727
TNM stage (III vs IV)	1.455 (1.021-2.074)	0.038		0.991 (0.646-1.518)	0.965
Uric acid (>213 μmol/L vs ≤213 μmol/L)	0.617 (0.424-0.900)	0.012		0.850 (0.566-1.276)	0.434
NLR (≤4.86 vs >4.86)	0.570 (0.387-0.842)	0.005		0.712 (0.473-1.074)	0.106
CYFRA21-1 (>2.35 ng/mL vs ≤2.35 ng/mL)	1.651 (1.109-2.459)	0.013		1.677 (1.112-2.530)	0.014
LDH (≤245 U/L vs >245 U/L)	2.044 (1.459-2.866)	<0.001		2.205 (1.549-3.139)	<0.001
TFA (Yes vs No)	3.645 (2.558-5.193)	<0.001		3.286 (2.228-4.846)	<0.001
HR: hazard ratio.					

## 3 讨论

近年来，肿瘤免疫治疗取得巨大进展，为晚期肺癌患者带来生存获益。免疫治疗联合化疗已经获批成为晚期NSCLC的一线治疗方案^[11]^。目前，在我国临床上选用最多的ICIs为PD-1/PD-L1抑制剂，包含帕博利珠单抗、信迪利单抗、卡瑞利珠单抗、特瑞普利单抗、阿替利珠单抗等多种药物；受限于价格和医保政策，本研究中，186例（93.0%）患者应用PD-1单抗，仅14例（7.0%）应用PD-L1单抗，其中以卡瑞利珠单抗应用最多，为118例（59.0%）。PD-1是一种表达于多种免疫细胞表面的免疫抑制分子^[12]^，当肿瘤细胞表面表达的PD-L1与PD-1结合时，便会向T细胞传递负向调控信号，导致T细胞无法识别癌细胞，介导肿瘤免疫逃避^[13]^，导致肿瘤的发生与发展。上述免疫抑制性通路可以被ICIs阻断，使细胞毒性T细胞恢复活性，从而起到识别和杀伤肿瘤细胞的作用。TFA是常见的免疫相关不良内分泌事件，主要表现为甲状腺功能减退、甲状腺功能亢进和一过性甲状腺炎^[14]^，其发生机制尚未完全阐明，可能的机制为恢复活性的细胞毒性T细胞除了识别肿瘤抗原外，还能识别正常的自身抗原^[15]^。Yamauchi等^[16]^的研究发现可以在正常甲状腺组织中检测到PD-L1和PD-L2的基因表达。表达PD-1的淋巴细胞和表达PD-L1/PD-L2的甲状腺细胞之间相互作用，可以抑制T细胞从而抑制自身免疫反应，达到保护甲状腺组织的作用^[17]^，而ICIs可以阻断这种相互作用从而诱导甲状腺细胞向自身反应性T和B淋巴细胞浸润，最终导致TFA的发生。

本研究发现，与甲状腺功能正常组相比，TFA组患者的PFS显著延长，中位PFS为19.0个月，ORR及DCR也显著改善。提示TFA可能是一个预测免疫治疗预后的生物标志物。原因可能为当发生TFA时，表达PD-1的淋巴细胞与表达PD-L1/PD-L2的甲状腺细胞之间相互作用被阻断，表明可能会有更多细胞受到ICIs的作用，代表机体对ICIs的免疫反应增强，从而获得更好的疗效。我们对不同治疗组进行分析，发现单药免疫组预后最好，原因可能为选用单药治疗的患者PD-L1表达水平较高或肿瘤负荷较小，但仅有8例患者应用单药免疫治疗，可能存在个体异质性及误差。本研究同时发现ECOG PS评分0分-1分、LDH≤245 U/L、CYFRA21-1>2.35 ng/mL与较长的PFS有关，是预后的影响因素。血清LDH水平被证明是肿瘤负荷大的肿瘤组织缺氧的间接指标，LDH水平可以评估患者的肿瘤负荷及炎症状态^[18]^；ECOG PS用以评估肿瘤患者的一般活动状态^[19]^；CYFRA21-1是一种肺癌相关的肿瘤标志物，对于肺癌的诊断、病情监测、疗效评价具有一定的应用价值，可以有效评估NSCLC患者的预后^[20]^。Zhang等^[21]^发现，CYFRA21-1是肺腺癌预后的独立危险因素。PD-L1表达情况可以用来预测免疫治疗疗效^[22]^，分析本研究中PD-L1表达阳性和阴性的患者，发现表达阳性患者的中位PFS优于表达阴性患者，但是差异无统计学意义，原因可能为本研究中PD-L1表达结果缺失患者较多，且样本量不足。既往也有研究发现肿瘤突变负荷^[23]^和微卫星不稳定^[24]^可以用来预测免疫治疗的疗效，但由于价格和取材等问题，应用价值有限。

本研究回顾性分析了200例一线接受免疫治疗的晚期NSCLC患者，TFA的发生率为43.0%，高于部分既往研究^[25,26]^，原因可能为本研究纳入的均为一线接受免疫治疗的患者，我们可能低估了一线应用免疫治疗的患者TFA的发生率，且本研究随访甲状腺功能的时间较长，我们推测随着时间的推移患者发生TFA的概率可能也会升高。本研究同时发现ECOG PS、胸腔积液、LDH可能是TFA发生的影响因素。ECOG PS评分高的患者身体状况较差，机体的免疫功能低下，从而影响TFA的发生。甲状腺功能减退等多种甲状腺疾病都可能继发胸腔积液，合并胸腔积液的患者可能也有潜在的机制来影响TFA的发生，未来仍需进一步的研究来证实。本研究86例TFA患者中，严重程度为1级的有66例（76.7%），2级有20例（23.3%），未观察到2级以上的TFA发生。TFA发生的中位时间是6.4个月，结合甲状腺功能简便易测，因此我们推荐的甲状腺功能监测方法是在首次接受ICIs前测定TSH、FT3、FT4，并在接受免疫治疗后的至少前6个月内每次应用ICIs前均行甲状腺功能测定，必要时应在免疫治疗期间持续监测甲状腺功能，尽早发现可能存在的TFA并及时作出干预。本研究也存在不足之处：作为单中心回顾性研究，存在信息偏倚可能，且随访时间较短，暂未收集到患者完整的生存期数据。

综上所述，本研究通过对真实世界的数据分析，发现TFA可能是免疫治疗疗效的影响因素，接受免疫治疗后发生TFA的晚期NSCLC患者可能有更好的预后，并且本研究发现了ECOG PS、胸腔积液、LDH可能是影响TFA发生的影响因素，未来仍需大样本、多中心的临床试验来证实。

## References

[1] Zheng R, Zhang S, Zeng H, et al. Cancer incidence and mortality in China, 2016. J Nat Cancer Cent, 2022, 2(1): 1-9. doi: 10.1016/j.jncc.2022.02.002 PMC11256658 39035212

[2] Siegel RL, Miller KD, Fuchs HE, et al. Cancer statistics, 2022. CA Cancer J Clin, 2022, 72(1): 7-33. doi: 10.3322/caac.21708 35020204

[3] Le Burel S, Champiat S, Mateus C, et al. Prevalence of immune-related systemic adverse events in patients treated with anti-programmed cell death 1/anti-programmed cell death-ligand 1 agents: A single-centre pharmacovigilance database analysis. Eur J Cancer, 2017, 82: 34-44. doi: 10.1016/j.ejca.2017.05.032 28646772

[4] Michot JM, Bigenwald C, Champiat S, et al. Immune-related adverse events with immune checkpoint blockade: a comprehensive review. Eur J Cancer, 2016, 54: 139-148. doi: 10.1016/j.ejca.2015.11.016 26765102

[5] Thompson JA, Schneider BJ, Brahmer J, et al. Management of immunotherapy-related toxicities, version 1. 2022, NCCN Clinical Practice Guidelines in Oncology. J Natl Compr Canc Netw, 2022, 20(4): 387-405. doi: 10.6004/jnccn.2022.0020 35390769

[6] Barroso-Sousa R, Barry WT, Garrido-Castro AC, et al. Incidence of endocrine dysfunction following the use of different immune checkpoint inhibitor regimens: A systematic review and meta-analysis. JAMA Oncol, 2018, 4(2): 173-182. doi: 10.1001/jamaoncol.2017.3064 28973656 PMC5838579

[7] Wu L, Xu Y, Wang X, et al. Thyroid dysfunction after immune checkpoint inhibitor treatment in a single-center Chinese cohort: a retrospective study. Endocrine, 2023. doi: 10.1007/s12020-023-03323-9 36867366

[8] von Itzstein MS, Gonugunta AS, Wang Y, et al. Divergent prognostic effects of pre-existing and treatment-emergent thyroid dysfunction in patients treated with immune checkpoint inhibitors. Cancer Immunol Immunother, 2022, 71(9): 2169-2181. doi: 10.1007/s00262-022-03151-2 35072744 PMC9308834

[9] Lima Ferreira J, Costa C, Marques B, et al. Improved survival in patients with thyroid function test abnormalities secondary to immune-checkpoint inhibitors. Cancer Immunol Immunother, 2021, 70(2): 299-309. doi: 10.1007/s00262-020-02664-y 32712715 PMC10991153

[10] Eisenhauer EA, Therasse P, Bogaerts J, et al. New response evaluation criteria in solid tumours: revised RECIST guideline (version 1.1). Eur J Cancer, 2009, 45(2): 228-247. doi: 10.1016/j.ejca.2008.10.026 19097774

[11] Paz-Ares L, Vicente D, Tafreshi A, et al. A randomized, placebo-controlled trial of pembrolizumab plus chemotherapy in patients with metastatic squamous NSCLC: protocol-specified final analysis of KEYNOTE-407. J Thorac Oncol, 2020, 15(10): 1657-1669. doi: 10.1016/j.jtho.2020.06.015 32599071

[12] Ahmadzadeh M, Johnson LA, Heemskerk B, et al. Tumor antigen-specific CD 8 T cells infiltrating the tumor express high levels of PD-1 and are functionally impaired. Blood, 2009, 114(8): 1537-1544. doi: 10.1182/blood-2008-12-195792 19423728 PMC2927090

[13] Dobosz P, Stępień M, Golke A, et al. Challenges of the immunotherapy: perspectives and limitations of the immune checkpoint inhibitor treatment. Int J Mol Sci, 2022, 23(5): 2847. doi: 10.3390/ijms23052847 35269988 PMC8910928

[14] Lee H, Hodi FS, Giobbie-Hurder A, et al. Characterization of thyroid disorders in patients receiving immune checkpoint inhibition therapy. Cancer Immunol Res, 2017, 5(12): 1133-1140. doi: 10.1158/2326-6066.CIR-17-0208 29079654 PMC5748517

[15] Chalan P, Di Dalmazi G, Pani F, et al. Thyroid dysfunctions secondary to cancer immunotherapy. J Endocrinol Invest, 2018, 41(6): 625-638. doi: 10.1007/s40618-017-0778-8 29238906 PMC5953760

[16] Yamauchi I, Sakane Y, Fukuda Y, et al. Clinical features of Nivolumab-induced thyroiditis: A case series study. Thyroid, 2017, 27(7): 894-901. doi: 10.1089/thy.2016.0562 28537531

[17] Zhan L, Feng HF, Liu HQ, et al. Immune checkpoint inhibitors-related thyroid dysfunction: epidemiology, clinical presentation, possible pathogenesis, and management. Front Endocrinol (Lausanne), 2021, 12: 649863. doi: 10.3389/fendo.2021.649863 PMC8224170 34177799

[18] Svaton M, Blazek J, Krakorova G, et al. Laboratory parameters are possible prognostic markers in patients with advanced-stage NSCLC treated with bevacizumab plus chemotherapy. J Cancer, 2021, 12(19): 5753-5759. doi: 10.7150/jca.58851 34475989 PMC8408121

[19] Ready NE, Audigier-Valette C, Goldman JW, et al. First-line nivolumab plus ipilimumab for metastatic non-small cell lung cancer, including patients with ECOG performance status 2 and other special populations: CheckMate 817. J Immunother Cancer, 2023, 11(2): e006127. doi: 10.1136/jitc-2022-006127 PMC9896179 36725084

[20] Ma K, Wang H, Jiang X, et al. Prognostic value of combination of controlling nutritional status and tumor marker in patients with radical non-small-cell lung cancer. Dis Markers, 2022, 2022: 4764609. doi: 10.1155/2022/4764609 PMC9525734 36193507

[21] Zhang L, Liu D, Li L, et al. The important role of circulating CYFRA21- 1 in metastasis diagnosis and prognostic value compared with carcinoembryonic antigen and neuron-specific enolase in lung cancer patients. BMC Cancer, 2017, 17(1): 96. doi: 10.1186/s12885-017-3070-6 PMC5290605 28152979

[22] Chen K, Cheng G, Zhang F, et al. PD-L1 expression and T cells infiltration in patients with uncommon EGFR-mutant non-small cell lung cancer and the response to immunotherapy. Lung Cancer, 2020, 142: 98-105. doi: 10.1016/j.lungcan.2020.02.010 32120230

[23] Cheng Y, Zhang Y, Yuan Y, et al. The comprehensive analyses of genomic variations and assessment of TMB and PD-L 1 expression in Chinese lung adenosquamous carcinoma. Front Genet, 2021, 11: 609405. doi: 10.3389/fgene.2020.609405 33679868 PMC7925901

[24] Pecci F, Cantini L, Bittoni A, et al. Beyond microsatellite instability: evolving strategies integrating immunotherapy for microsatellite stable colorectal cancer. Curr Treat Options Oncol, 2021, 22(8): 69. doi: 10.1007/s11864-021-00870-z 34110510 PMC8192371

[25] Zhong X, Ying J, Liao H, et al. Association of thyroid function abnormality and prognosis in non-small-cell lung cancer patients treated with PD-1 inhibitors. Future Oncol, 2022, 18(18): 2289-2300. doi: 10.2217/fon-2021-1537 35440175

[26] Zhou Y, Xia R, Xiao H, et al. Thyroid function abnormality induced by PD-1 inhibitors have a positive impact on survival in patients with non-small cell lung cancer. Int Immunopharmacol, 2021, 91: 107296. doi: 10.1016/j.intimp.2020.107296 33360368

